# Supplementary material for: Early Life Polychlorinated Biphenyl 126 Exposure Disrupts Gut Microbiota and Metabolic Homeostasis in Mice Fed with High-Fat Diet in Adulthood
Source: Metabolites. 2022 Sep 23;12(10):894. doi: 10.3390/metabo12100894 (PMC9609008; doi:10.3390/metabo12100894)
Supplement: Supplementary file 1 [file metabolites-12-00894-s001.zip › metabolites-1904564-supplementary.pdf]

Early Life Polychlorinated Biphenyl 126 Exposure Disrupts Gut Microbiota and Metabolic Homeostasis in Mice Fed with High-Fat Diet in Adulthood

Yuan Tian [1], Bipin Rimal [1], Wei Gui [2], Imhoi Koo [1], Philip B. Smith [2], Shigetoshi Yokoyama [1] and Andrew D. Patterson [1,*]

[1] Department of Veterinary and Biomedical Sciences, The Pennsylvania State University, University Park, PA 16802, USA
[2] Huck Institutes of the Life Sciences, The Pennsylvania State University, University Park, PA 16802, USA
* Correspondence: adp117@psu.edu

*To whom correspondence should be addressed. Email: adp117@psu.edu. Address: 322 Life Science Bldg, University Park, PA 16802, Phone: 814-867-4565

Table S1. Composition of control diet (CTRL) F4031 and high fat diet (HFD) F3282.

**Component (%)**[1]	**CTRL**	**HFD**
Protein	20.5	20.5
Fat	7.2	36
Fiber	0	0
Ash	3.5	3.5
Moisture	<10	<10
Carbohydrate	61.6	35.7

[1]These components are reported on a % dry matter basis.

Table S2. mRNA gene-targeted primers used in this study

Gene	Abbreviation	Sequence (5'-3')
Cytochrome P450, family 1, member A1	Cyp1a1	CTCTTCCCTGGATGCCTTGAA<br>GGATGTGGCCCTTCTCAAATG
Cytochrome P450, family 1, member A2	Cyp1a2	GCCCCTGCCCTTCAGTGGTACAG<br>AGGAGTGGAGCCGATGCGGA
Cytochrome P450, family 1, member B1	Cyp1b1	GTCTGTGAATCATGACCCAGC<br>ACAGTTCCTCACCGATGCAC
NAD(P)H Quinone Dehydrogenase 1	Nqo1	TTCTGTGGCTTCCAGGTCTT<br>AGGCTGCTTGGAGCAAAATA
Tumor necrosis factor alpha	Tnf	GATCTCAAAGACAACCAACATGTG<br>CTCCAGCTGGAAGACTCCTCCCAG
Interleukin 1β	Il1ß	CCTTCCAGGATGAGGACATGA<br>TGAGTCACAGAGGATGGGCTC
Interleukin 6	Il6	GAGGATACCACTCCCAACAGACC<br>AAGTGCATCATCGTTGTTCATACA
Monocyte chemoattractant protein-1	Mcp1	TGATCCCAATGAGTAGGCTGGAG<br>ATGTCTGGACCCATTCCTTCTTG
Regulated on activation, normal T expressed and secreted	Rantes	CCAATCTTGCAGTCGTGTTTGT<br>CATCTCCAAATAGTTGATGTATTCTTG AAC
Prostaglandin-endoperoxide synthase 2	Ptgs2	GCTTCAAACAGTTTCTCTACAACAA<br>CATTTCTTCCCCCAGCAAC
Stearoyl-CoA desaturase-1	Scd1	TTCTTGCGATACACTCTGGTGC<br>CGGGATTGAATGTTCTTGTCGT
Cluster of differentiation 36	Cd36	TGGCCTTACTTGGGATTGG<br>CCAGTGTATATGTAGGCTCATCCA
Acetyl-CoA carboxylase alpha	Acaca	TAACAGAATCGACACTGGCTGGCT<br>ATGCTGTTCCTCAGGCTCACATCT
Carnitine palmitoyltransferase 1A	Cpt1a	CGTGACGTTGGAATC<br>TCTGCGTTTATGCCTATC
Diacylglycerol O-Acyltransferase 2	Dgat2	CGCAGCGAAAACAAGAATAA<br>GAAGATGTCTTGGAGGGCTG
Fatty acid synthase	Fasn	GGTGTGGTGGGTTTGGTGAATTGT<br>TCACGAGGTCATGCTTTAGCACCT
Phosphoenolpyruvate carboxykinase	Pepck	GGCCACAGCTGCTGCAG<br>GGTCGCATGGCAAAGGG

Glucose-6-phosphatase	G6pase	CTGTGAGACCGGACCAGGA<br>GACCATAACATAGTATACACCTGCTGC
Glucokinase	Gck	CAACTGGACCAAGGGCTTCAA<br>TGTGGCCACCGTGTCATTC
Pyruvate carboxylase	Pcx	GGACTCCTTTGGACACAGAG<br>AATCTCATTCTCATACACGTCGG
Phosphoenolpyruvate carboxykinase 1	Pck1	CCATCCCAACTCGAGATTCTG<br>CTGAGGGCTTCATAGACAAGG
Fructose-1,6-bisphosphatase 1	Fbp1	CTGATATTCACCGCACTCTGG<br>CGGCCTTCTCCATGACATAAG
Glyceraldehyde-3-phosphate dehydrogenase	Gapdh	CCTCGTCCCGTAGACAAAATG<br>TGAAGGGGTCGTTGATGGC

Table S3. Serum cytokine (32-Plex) assay results.

Unit: pg/mL	CTRL				HFD		
	Vehicle	PCB 126	P value		Vehicle	PCB 126	P value
**Eotaxin**	1889.0 ± 354.9[1]	1710.5 ± 660.5	0.57[2]		1484.8 ± 271.9	1422.3 ± 704.0	0.85
**G-CSF[3]**	689.6 ± 234.6	842.8 ± 477.3	0.50		587.4 ± 276.0	803.2 ± 366.5	0.28
**GM-CSF**	38.5 ± 22.5	36.8 ± 16.1	0.93		37.2 ± 40.6	29.9 ± 6.3	0.75
**IFNγ**	3.85 ± 1.88	4.70 ± 2.94	0.60		8.35 ± 8.45	3.40 ± 1.56	0.21
**IL-1α**	1087.1 ± 666.0	1342.3 ± 1088.2	0.64		428.1 ± 160.3	468.8 ± 704.1	0.90
**IL-1β**	14.8 ± 6.2	31.7 ± 24.5	0.20		23.8 ± 10.5	24.6 ± 22.7	0.94
**IL-2**	20.0 ± 10.2	17.6 ± 7.2	0.66		26.8 ± 18.7	12.5 ± 6.4	0.12
**IL-3**	3.09 ± 1.75	3.40 ± 0.67	0.75		1.93 ± 0.89	1.43 ± 0.98	0.60
**IL-4**	1.27 ± 0.67	1.25 ± 0.52	0.96		1.30 ± 0.84	2.33 ± 1.95	0.32
**IL-5**	18.0 ± 4.6	16.4 ± 3.2	0.49		24.1 ± 10.2	26.2 ± 8.2	0.69
**IL-6**	8.25 ± 3.47	7.24 ± 3.10	0.61		15.7 ± 5.1	16.0 ± 9.1	0.94
**IL-7**	16.7 ± 6.7	13.5 ± 4.3	0.38		22.7 ± 14.1	11.6 ± 5.1	0.12
**IL-9**	53.5 ± 13.6	45.4 ± 10.1	0.29		46.6 ± 8.2	60.7 ± 23.1	0.21
**IL-10**	22.0 ± 11.3	18.2 ± 14.1	0.67		21.1 ± 10.4	22.6 ± 12.8	0.83
**IL-12p40**	58.6 ± 19.2	52.3 ± 17.7	0.57		55.8 ± 23.7	42.3 ± 17.2	0.32
**IL-12p70**	52.5 ± 26.6	50.7 ± 32.1	0.93		47.9 ± 27.7	69.7 ± 40.3	0.37
**IL-13**	95.5 ± 16.1	88.0 ± 28.9	0.62		90.8 ± 23.4	79.4 ± 13.1	0.33
**IL-15**	158.5 ± 71.8	190.1 ± 106.5	0.57		209.4 ± 117.5	169.1 ± 111.4	0.56
**IL-17**	34.9 ± 12.1	28.4 ± 8.7	0.31		35.9 ± 18.7	34.0 ± 14.3	0.85

**IP-10**	236.3 ± 29.2	226.4 ± 29.0	0.57		321.7 ± 90.0	248.8 ± 68.4	0.15
**KC**	198.3 ± 47.6	183.1 ± 103.0	0.77		268.2 ± 188.6	184.3 ± 90.5	0.36
**LIF**	2.23 ± 1.81	3.87 ± 4.68	0.45		1.94 ± 0.86	0.94 ±0.48	**0.04**
**LIX**	3478 ± 895	4129 ± 1736	0.44		4909 ± 2654	12440 ± 4517	**0.01**
**MCP-1**	106.9 ± 30.5	96.8 ± 19.2	0.52		140.6 ± 29.1	115.0 ± 22.0	0.12
**M-CSF**	31.6 ± 10.6	29.9 ± 16.0	0.84		28.8 ± 18.2	22.1 ± 11.0	0.47
**MIG**	950.6 ± 324.2	855.9 ± 355.1	0.64		1346.1 ± 704.3	933.8 ± 693.5	0.33
**MIP-1α**	148.7 ± 25.6	150.0 ± 55.5	0.96		161.5 ± 51.7	140.9 ± 46.0	0.50
**MIP-1B**	203.4 ± 65.3	118.9 ± 71.9	0.08		155.1 ± 65.5	79.1 ± 79.3	0.10
**MIP-2**	377.7 ± 92.5	425.4 ± 285.6	0.71		283.5 ± 118.9	181.1 ± 103.1	0.19
**RANTES**	74.1 ± 37.8	94.7 ± 30.4	0.34		59.6 ± 18.5	84.6 ± 22.1	0.06
**TNFα**	21.3 ± 5.9	22.0 ± 7.2	0.85		24.4 ± 5.9	22.1 ± 7.9	0.59
**VEGF**	1.97 ± 0.50	2.01 ± 0.49	0.88		2.06 ± 0.86	1.62 ± 0.88	0.41

[1]Values are means ± S.D. (n = 6 per group).

[2]Two-tailed unpaired t test analyses were performed.

[3]G-CSF: granulocyte colony stimulating factor; GM-CSF: Granulocyte/macrophage colony stimulating factor; IFNγ: interferon gamma; IL: interleukin; IP-10: interferon gamma inducible protein 10; KC: keratinocyte chemoattractant; LIF: leukaemia inhibitory factor; LIX: lipopolysaccharide-induced CXC chemokine; MCP-1: monocyte chemoattractant protein-1; M-CSF: macrophage colony-stimulating factor; MIG: monokine induced by IFN-γ; MIP: macrophage inflammatory protein; RANTES:

regulated upon activation, normal T cell expressed and presumably secreted; TNFα: tumor necrosis factor alpha; VEGF: vascular endothelial growth factor.

Table S4. Relative abundances of significantly changed genus in mice fed with CTRL.

Genus	Phylum	Vehicle	PCB 126	P value
Lacrimispora	*Firmicutes*	0.48 ± 0.06[1]	0.36 ± 0.03	0.002[2]
Anaerocolumna	*Firmicutes*	0.34 ± 0.04	0.25 ± 0.03	0.003
Anaerobutyricum	*Firmicutes*	0.23 ± 0.03	0.17 ± 0.02	0.004
Anaerostipes	*Firmicutes*	0.46 ± 0.05	0.37 ± 0.03	0.007
Mediterraneibacter	*Firmicutes*	0.39 ± 0.06	0.30 ± 0.03	0.008
Blautia	*Firmicutes*	2.89 ± 0.35	2.31 ± 0.26	0.01
Butyrivibrio	*Firmicutes*	0.41 ± 0.05	0.33 ± 0.04	0.01
Bacillus	*Firmicutes*	0.38 ± 0.04	0.32 ± 0.04	0.02
Clostridioides	*Firmicutes*	0.78 ± 0.10	0.61 ± 0.10	0.02
Hungatella	*Firmicutes*	0.41 ± 0.04	0.35 ± 0.04	0.03
Eubacterium	*Firmicutes*	0.46 ± 0.05	0.39 ± 0.06	0.04
Lachnoclostridium	*Firmicutes*	7.54 ± 1.11	6.16 ± 0.82	0.04
Ruthenibacterium	*Firmicutes*	0.35 ± 0.04	0.28 ± 0.06	0.04
Roseburia	*Firmicutes*	1.20 ± 0.17	1.00 ± 0.11	0.04
Streptococcus	*Firmicutes*	0.41 ± 0.06	0.34 ± 0.04	0.04
Caproiciproducens	*Firmicutes*	0.20 ± 0.02	0.17 ± 0.02	0.04
Akkermansia	*Verrucomicrobia*	11.18 ± 4.92	21.79 ±5.37	0.004

[1]These are percent relative abundances. Values are means ± S.D. (n = 6 per group).

[2]Two-tailed Unpaired t test analyses were performed.

Table S5. Relative abundances of significantly changed genus in mice fed with HFD.

Genus	Phylum	Vehicle	PCB 126	P value
Muribaculum	*Bacteroidetes*	0.025 ± 0.007[1]	0.016 ± 0.005	0.02[2]
Duncaniella	*Bacteroidetes*	0.018 ± 0.005	0.011 ± 0.003	0.02
Romboutsia	*Firmicutes*	0.012 ± 0.004	0.018 ± 0.004	0.04
Bacteroides	*Bacteroidetes*	0.011 ± 0.002	0.007 ± 0.002	0.02
Adlercreutzia	*Actinobacteria*	0.002 ± 0.001	0.004 ± 0.002	0.04
Prevotella	*Bacteroidetes*	0.003 ± 0.001	0.002 ± 0.001	0.03
Parabacteroides	*Bacteroidetes*	0.002 ± 0.000	0.001 ± 0.0000	0.02

[1]These are percent relative abundances. Values are means ± S.D. (n = 6 per group).

[2] Two-tailed unpaired t test analyses were performed.

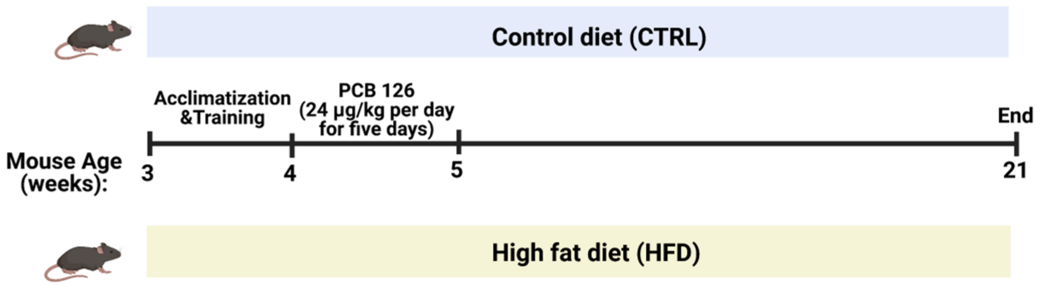

Figure S1. Experimental schedule of the present study.

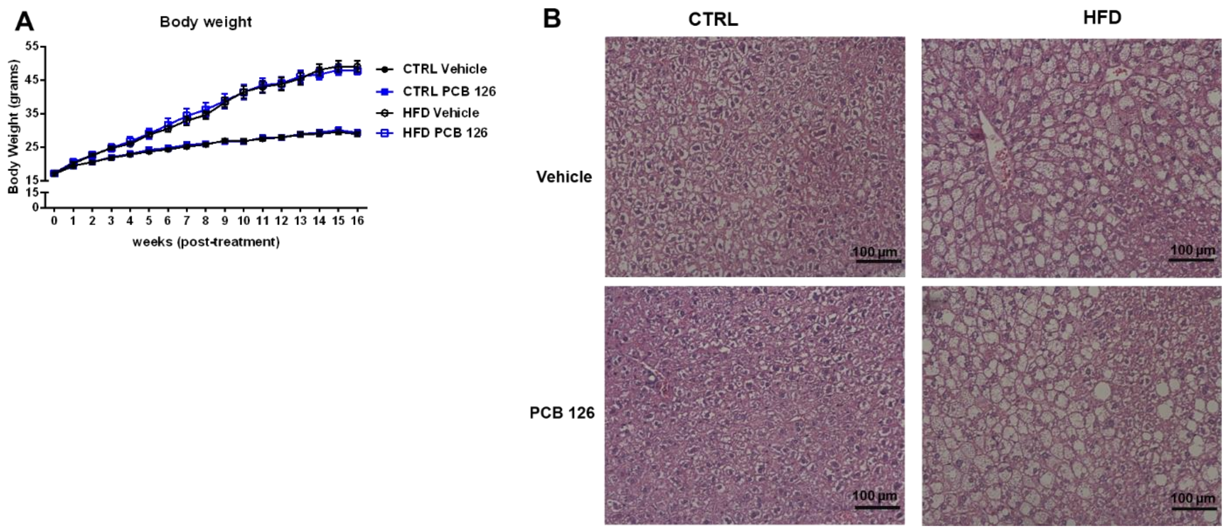

Figure S2. (A) Body weight of mice recorded every week from CTRL or HFD-fed mice with vehicle or PCB 126 exposure. (B) Light microscopic examination of H&E-stained liver sections from CTRL or HFD-fed mice with vehicle or PCB 126 exposure. Values are means ± S.D. (n = 6 per group).

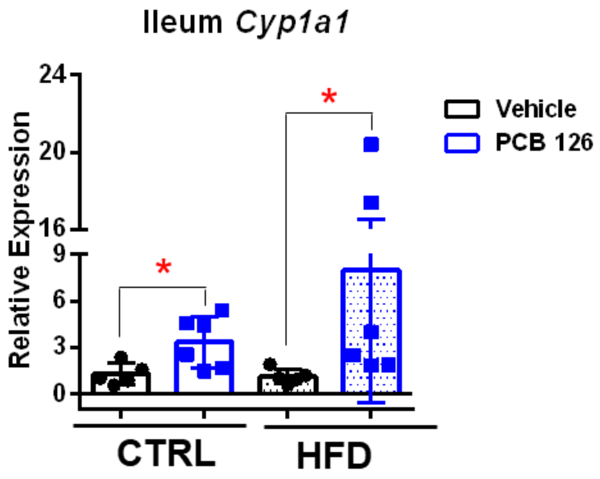

Figure S3. qPCR analysis of mRNA levels of AHR targeted gene in the ileum from CTRL or HFD-fed mice with vehicle or PCB 126 exposure. Values are means ± S.D. (n = 6 per group). * $p < 0.05$ compared to vehicle

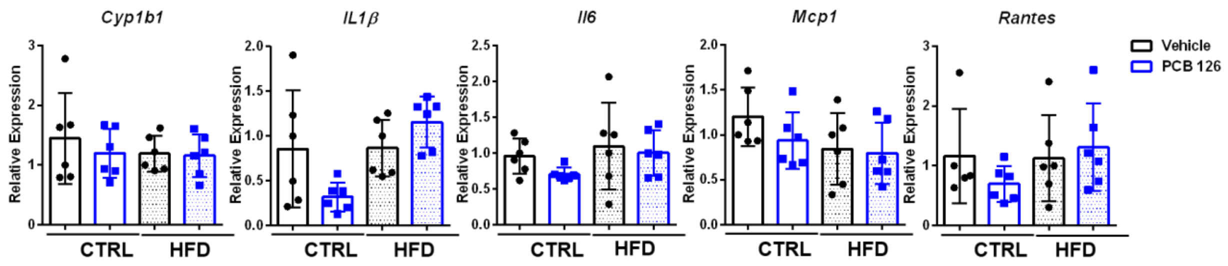

Figure S4. qPCR analysis of mRNA levels of inflammatory cytokines in the adipose from CTRL or HFD-fed mice with vehicle or PCB 126 exposure. Values are means ± S.D. (n = 6 per group).

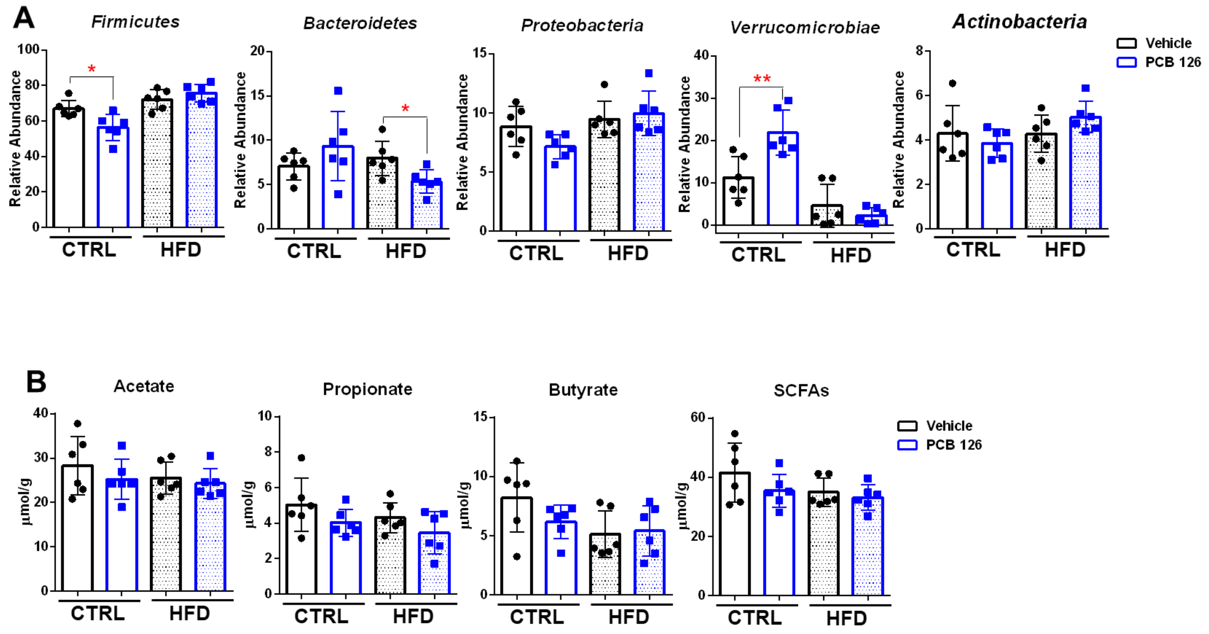

Figure S5. (A) Relative abundance of cecal bacteria from phylum in cecal content from CTRL or HFD-fed mice with vehicle or PCB 126 exposure. (B) NMR analysis of cecal short-chain fatty acids (SCFAs) from CTRL or HFD-fed mice with vehicle or PCB 126 exposure. Values are means ± S.D. (n = 6 per group). * $p < 0.05$, ** $p < 0.01$ compared to vehicle

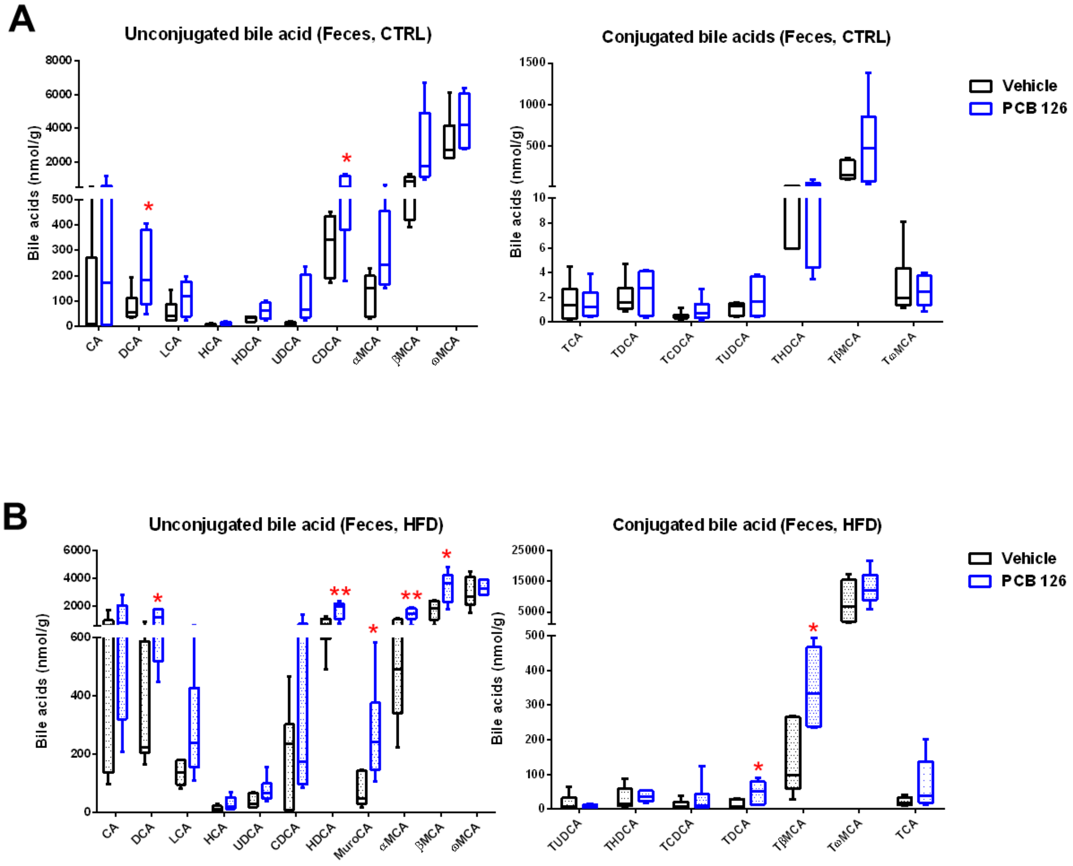

Figure S6. (A-B) Quantitative UPLC-MS analysis of bile acids in the feces from CTRL (A) or HFD-fed (B) mice with vehicle or PCB 126 exposure. Values are median and interquartile range (n = 6 per group). * $p < 0.05$, ** $p < 0.01$ compared to vehicle. CA, cholic acid; DCA, deoxycholic acid; LCA, lithocholic acid; UDCA, ursodeoxycholic acid; CDCA, chenodeoxycholic acid; HDCA, hydeoxycholic acid; MuroCA, murocholic acid; MCA, muricholic acid; T, taurine-conjugated species.
